# The Role of Sports Club Participation on Stability of Motor Performance and Body Composition: A Longitudinal Study in Primary School Children

**DOI:** 10.1155/2024/2952520

**Published:** 2024-06-10

**Authors:** Andreas Speer, Alexandra Ziegeldorf, Heike Streicher, Hagen Wulff, Petra Wagner

**Affiliations:** Faculty of Sport Science Leipzig University, Leipzig, Germany

## Abstract

**Methods:**

MP and BC of 295 children (161 girls) with a mean age of 8.42 ± 0.36 years were measured annually with the German Motor Test 6-18. Based on self-reports, children were divided into three groups according to consistent (CON), partial (PAR), and nonparticipation (NO) in SC. NO and PAR were then combined into NO-PAR. The stability of MP and BC was determined using Pearson's correlation coefficient (*r*). Associations of SC participation, MP, and BC were examined using robust mixed-model ANOVA (mmANOVA) additionally with first grade as covariate (ANCOVA).

**Results:**

More girls (39%) than boys (25%) were classified in PAR. The stability of MP (*r* = .755) and BMI (*r* = .889) was moderately high. Children in CON (*r* = .847) showed lower stability in BMI than NO-PAR (*r* = .923). mmANOVA revealed better overall MP for both sexes in CON except for balance and BMI. Boys in CON showed better performance in flexibility, endurance, and speed than NO-PAR. ANCOVA confirmed unadjusted results for boys.

**Conclusion:**

Lower stability of BMI due to CON indicates a greater variance in ranking and thus the chance to adjust weight management in childhood. Girls' MP benefited less from SC participation, which may be due to their more frequent rates in PAR. Therefore, interventions to increase engagement in SC should focus on a stepwise approach from none to partial and finally to long-term participation.

## 1. Introduction

The concept of motor performance (MP) is based on a structural model that includes coordination-related and conditional-related motor capacities, which are referred to as motor abilities [1]. Apart from differences in theoretical approach but similarities in components and especially in assessment [2], the term physical fitness (PF) reflects another widely accepted approach that refers to a person's ability to perform physical work [3]. Following these considerations, the basic motor abilities include endurance, strength, speed, coordination, and flexibility [1, 4] and also illustrate skill-related and health-related fitness components [5]. However, these components play a key role in understanding and influencing health [3, 6].

Even in childhood and adolescence, a cardiorespiratory fitness level above a critical threshold reduces the risk of cardiovascular disease [7]. Furthermore, children's fundamental motor skills are positively related to physical activity (PA) and inversely related to weight status [8] and impact the capability to be physically active and promote health across the lifespan [9]. In the context of longitudinal observation of MP, the concept of tracking covers the maintenance of an individual's rank or position within a distribution of values of population over time [10, 11]. It also includes the predictability of future values through early measurements. As a result of tracking analyses, maintaining the same rank over time means stability of values, while changing the rank means instability [12]. For epidemiological research, tracking of MP may practically be important to evaluate how motor abilities are maintained and positively impact health later in life. This relevance can be broadened to the purpose of avoiding detrimental health outcomes such as disease or abnormal weight status [13]. For coaches, it can be crucial to know if the prerequisites to MP as a young talent are sustained for later peak performance.

Taking into account that tracking outcome mainly depends on the cohort of the target population and its developmental status at baseline and follow-up [10], current research across childhood has shown low to moderately high stability [14], or low-to-moderate stability [15] of fitness- and coordination-related MP. From childhood to adolescence, Sasayama and Adachi [16] found moderate-to-high stability, while True et al. [17] tracked PF components indicating low-to-moderate stability. Blasquez Shigaki et al. [18] reported moderate-to-high stability of PF in the transition from childhood to adulthood. Nonparametric tracking methods consistently showed lower stability of MP than parametric data analysis [13, 17, 18]. However, as a result of a meta-analysis, García-Hermoso et al. [19] indicated that tracking of cardiorespiratory fitness, muscle strength, and muscular endurance showed moderate stability from childhood and/or adolescence to adulthood independent of the used method and length of follow-up. Assessing the direction of rank change, Souza et al. [20] tracked individual position within PF performance groups categorized into tertiles (low, middle, and high) among young adolescents aged 10 to 12 years. With a varying sample size (*N* = 252 to 295), 1.6-7.2% of subjects changed their PF level from low to high, which was interpreted as positive instability, and 2.0-6.0% of children moved from high to low, which meant negative instability. This is consistent with Henrique et al. [15], who classified a cohort of 245 children (aged 6 to 9 years) comparably regarding to tracking of gross motor coordination. To identify key factors that influence the direction of the course of individual position across PF tertile groups, Werneck et al. [21] examined 372 elementary school children (9-12 years). They revealed that larger increases in body fat were associated with a higher likelihood of dropping to a lower PF tertile in boys (OR = 4.17) and with a lower likelihood of improving to a higher PF tertile in both sexes (OR = .25 − .37). From a public health perspective, there is a need to investigate on how children's PA affects the direction of change in PF ranking and how it contributes to variance explanation in tracking analyses [14].

As a subcategory of leisure-time PA [3], sports participation has been recommended as a key strategy to reduce inactivity and promote health [22]. In addition, participation in a sports club (SC), classified as organized sports participation [3], represents a crucial subdomain of PA that follows an ongoing trend in childhood [23]. Previous research in childhood showed that SC participants were more physically fit [24, 25] and had better exercise capacity, lower resting heart rate, and higher muscle mass than non-SC participants [26].

Despite this evidence, it is currently unclear whether SC participation affects childhood MP stability. Otherwise, further research could provide important information for initiating interventions to help children maintain or improve their MP at crucial time points. For a better understanding of the stability of MP and the role of SC participation in childhood, the following research questions will be explored:

- What is the prevalence of SC participation?
- How stable is MP in dependence of SC participation?
- Are changes in MP over time associated with SC participation?

## 2. Materials and Methods

### 2.1. Study Design

The examination was a part of the KOMPASS-2-study, a longitudinal observational study in Leipzig (Germany) covering four years (2014-2018) [27]. A total of 65 primary schools were invited to take part in the study. Due to organizational constraints and the limited use of sports facilities, 30 schools were finally included in the longitudinal observation. The participating schools were representative of the city in terms of children's social status and migration status. Following written consent from parents, data collection was conducted annually in each grade level. The study was approved by the ethics committee of the medical faculty of the University of Leipzig (approval number: 253-14-14072014). Missing values were imputed regarding recommendations of multiple data imputation [28].

### 2.2. Study Sample

A total of 1348 parents agreed to the annual data collection for each grade. Following that, children with cardiovascular or musculoskeletal diseases were omitted from the study. The total number of participants for each of the four measurement time points was 1140 (50.8% girls) in grade 1, 1135 (50.7% girls) in grade 2, 1019 (50.6% girls) in grade 3, and 1008 (50.0% girls) in grade 4. Descriptive statistics of the sample are shown in Table 1.

Not all participants could be retested across all measuring time points, e.g., due to acute injuries, absence of appropriate sportswear, transfer to a school not included in the recruitment, or voluntary withdrawal from the study. Those children who were not tested in each grade were not retained for further analysis considering the focus of the present study on longitudinal observations. Of the initial 1140 children who participated in the baseline measurement in grade 1 (Table 1), a total of 295 (25.9%) were tested at each measurement time point and represent the final longitudinal cohort for the present study (refer to Table 2). Girls and boys were equally distributed (*χ*^2^(1) = 2.47, *p* = .116) and matured from 6.93 ± 0.36 to 9.91 ± 0.38 years. Furthermore, children's body constitution changed from 1.24 ± 0.06 to 1.42 ± 0.06 m in height and from 24.86 ± 3.75 to 35.64 ± 7.02 kg in weight.

### 2.3. Data Collection and Processing

#### 2.3.1. Measuring MP and BC

To assess MP, the German Motor Test (Deutscher Motorik Test, DMT 6-18) was used [4]. The DMT 6-18 is a validated test battery and a widely accepted assessment tool for primary school-aged children [2]. It consists of eight MP subtests (20 m sprint, standing long jump, sit-ups, push-ups, 6-minute run, side jump, backward balancing, and stand and reach test) that indicate the MP level in speed, muscular strength, muscular endurance, cardiorespiratory endurance, agility, dynamic balance, and flexibility. In addition, basic anthropometric data (body weight, height) are recorded. The test stations were set up by trained sports students in the sports hall before a regular physical education (PE) lesson. According to the test manual [4], about 15 children completed an age-specific warm-up, followed by the 20 m sprint. Then, the children moved in pairs to a free test station. Finally, the 6 min run was performed together prior to the end of the lesson.

The raw values of the MP subtests were processed into sex- and age-standardized *z*-values using German reference data [4]. In addition, an overall MP score was calculated based on the mean of the eight *z*-values. A *z*-value of 100 corresponds to the average MP level in terms of age and sex of the reference data. Based on the measurement of body weight (kg) with a portable electronic scale (MC-780, Tanita Europe B.V., Netherlands) and height (m) with a stadiometer (seca 213, seca GmbH, Germany), the body mass index (BMI) was calculated (kg/m^2^). The BMI represents a widely used alternative indicator of body composition (BC) [29]. Finally, the raw values were converted into sex- and age-standardized BMI *z*-scores as in the MP procedure [4].

#### 2.3.2. Measuring SC Participation

SC participation was derived from an annual paper-pencil survey. Completing a questionnaire at home, parents were asked if their child was a member of a SC as part of a leisure-time PA. The filled out questionnaires were collected back at the school. For the entire sample (Table 1), the response rate was 76.8% at baseline (*N* = 875) and 33.3% in grade 4 (*N* = 380). Based on the annual dichotomous measure of SC membership (yes/no), children were divided into three longitudinal groups of SC participation. In line with Vandorpe et al. [30], children who had permanently joined a SC were classified as “consistently participating in SC” (CON). Conversely, children who had never joined a SC were classified as “no SC participation” (NO). Children who dropped out or commenced SC membership during primary school were classified as “partially participated in SC” (PAR).

### 2.4. Statistical Analysis

Descriptive statistics were calculated as mean and standard deviation for interval-scaled data and as frequency for nominal-scaled data. To analyze the prevalence of SC participation, a chi-square goodness-of-fit test was conducted. For the effect size, Cohen's *ω* was calculated (small: .10 ≤ *ω* < .30, medium: 30 ≤ *ω* < .50, and large: *ω* ≥ .50) [31]. To examine the relationship between SC participation and sex a 3 × 2 chi-square independence test was performed. The strength of relationship was assessed by Cramér's *V* (for interpretation refer to Cohen's *ω* above). In the case of a significant effect, pairwise comparisons among sex for each SC participation group were added.

To determine tracking of MP and its components, a mean Pearson's correlation coefficient (*r*) was calculated stepwise. First, all correlations between the four grades (six values in total) were computed and then converted into a Fisher's *z*-value [32]. The average correlation was then calculated and converted to a single mean *r*, as previously recommended. In addition, the associated 95% confidence intervals (CI) were calculated. If a CI does not contain the zero value, it can be assumed that there is a statistically significant correlation. Differences of *r* between the MP components were calculated using Fisher's *z*-test. According to Malina [10], the range of *r* is to be interpreted as follows: *r* < .30 (low), .30 ≤ *r* ≤ .60 (moderate), and *r* > .60 (moderately high).

To examine longitudinal associations of MP and BC across SC participation groups, a 3 × 4 mixed-model of analysis of variance (mmANOVA) was conducted. Due to unsatisfied assumptions regarding the homogeneity of variances, a robust method was calculated. In the case of significant main effects, MP and BC differences were examined via the Bonferroni-Holm adjusted post hoc tests. In the case of significant interaction effects, differences of MP and BC were examined via robust repeated measurement ANOVA separately for CON, NO, and PAR followed by post hoc analysis. For the effect size, the partial eta squared (*ƞ*^2^) was calculated (small: .010 ≤ *ƞ*^2^ < .060, medium: .060 ≤ *ƞ*^2^ < .140, and large: *ƞ*^2^ ≤ .140).

In addition, a robust analysis of covariance (ANCOVA) [33] was conducted to determine differences in MP and BC between SC participation groups controlling for baseline. In contrast to a standard ANCOVA, which tests the differences between the regression residuals of the independent variable considering the covariate, the robust ANCOVA compares the trimmed means of individual values (labeled as design points (DP)) along the smoothed regression lines [34]. The selection of five DPs is done automatically to cover a wide range of data for possible influences of baseline as a covariate. The setting of the smoothing parameter (*f* = 1.0 − 1.5) represented a compromise between overfitting while accounting for basic patterns in the data [34]. For Yuen's *t*-value, the trimmed mean difference of the DP with the largest distance between the regression lines and the adjusted 95% CI are reported. Cohen's *d* was used to estimate the effect size and then converted to *ƞ*^2^ [35] to provide a direct comparison with mmANOVA results. All data were analyzed using SPSS (version 29.0) and R (version 4.1.2). The significance level was set at *p* < .05.

## 3. Results

### 3.1. Prevalence of SC Participation

The majority of girls (48.4%) and boys (66.4%) were classified in CON (56.6%). Table 2 shows the distribution of frequencies of SC participation for the longitudinal sample.

Approximately one-third of children were classified in PAR (32.2%) and one-tenth of children were classified in NO (11.2%). The frequencies of the total sample differed statistically significant between the SC groups (*χ*^2^(2) = 91.47, *p* < .001, *ω* = .557), which meant a large effect according to Cohen [31]. Against a Bonferroni-adjusted significance level of *p*_adjusted_ = .008, the participation rate of CON (56.6) was higher than in PAR (32.2%, *χ*^2^(1) = 19.79, *p* < .001, *ω* = .275) and in NO (11.2%, *χ*^2^(1) = 89.78, *p* < .001, *ω* = .670). In addition, the participation rate of PAR was higher than that in NO (*χ*^2^(1) = 30.03, *p* < .001, *ω* = .484). Furthermore, results showed a significant relationship between sex and SC participation (*χ*^2^(2) = 9.64, *p* = .008, *V* = .181), which indicated a small effect. Post hoc analysis (*p*_adjusted_ = .007) revealed a significant difference with higher participation rates for girls (*N* = 62, 38.5%) than boys (*N* = 33, 24.6%) in PAR (*χ*^2^(1) = 8.85, *p* = .003, *ω* = .305). For CON (*χ*^2^(1) = .73, *p* = .395, *ω* = .066) and NO (*χ*^2^(1) = 2.46, *p* = .117, *ω* = .273), equal distributions were found in respect to sex.

### 3.2. Descriptive Statistics of MP and BC

**Figure 1:**
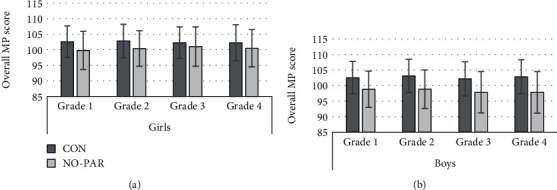
Means and standard deviations of overall MP score in SC participation and grade ((a) girls and (b) boys).

**Figure 2:**
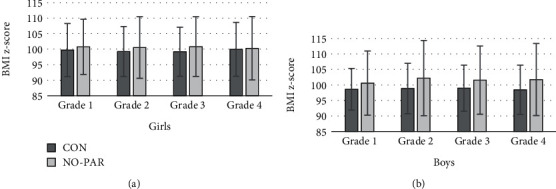
Means and standard deviations of BMI *z*-score in SC participation and grade ((a) girls and (b) boys).

To ensure sufficient statistical power for the analyses of associations between MP, BC, and SC participation, the small subsample of NO (girls: *N* = 21, boys: *N* = 12) was combined with PAR into a common group labeled NO-PAR.  Table 3 shows descriptive statistics of longitudinal observations of MP components. Figures 1 and 2 illustrate longitudinal data for overall MP score and BMI *z*-score.

### 3.3. Tracking of MP and BC

**Figure 3:**
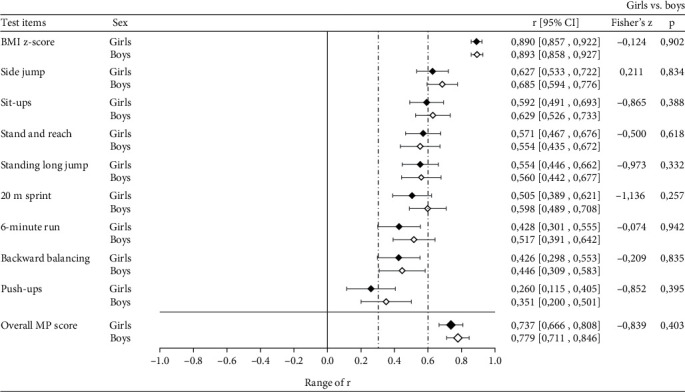
Mean Pearson's correlation coefficient (*r*) and 95% CI of MP across sex.

**Figure 4:**
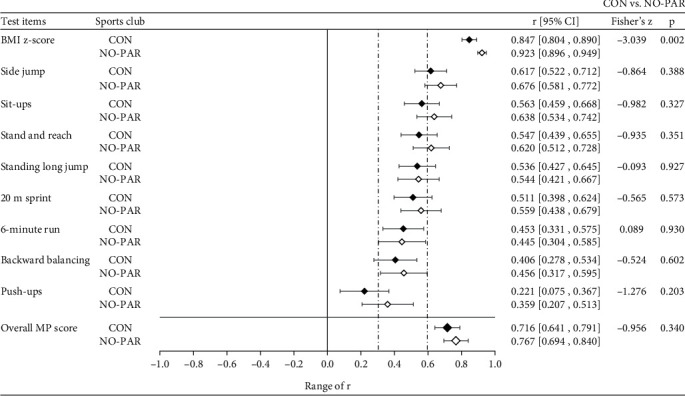
Mean Pearson's correlation coefficient (*r*) and 95% CI of MP across SC participation (CON = consistent SC participation; NO-PAR = no and partial SC participation).

All correlations were statistically significant with respect to the missing zero value within the 95% CI.  Table 4 shows the values of *r* for MP and BC. Forrest plots in Figures 3 and 4 illustrate the MP values of *r* in dependence of sex and SC participation, respectively. The vertical dashed lines illustrate the cut points for the strength of *r* according to Malina [10].

Overall MP was tracked with *r* = .755 (95% CI: .706, .804) and showed moderately high stability in primary school children. No difference was found between girls and boys (Fisher's *z* = −.839, *p* = .403) (Figure 3). Among the MP components, the values of *r* are widely ranged. The test item side jump showed the highest correlation of *r* = .652 (95% CI: .586, .718) and represented moderately high stability according to Malina [10]. In contrast, the lowest coefficient was found for push-ups (*r* = .302, 95% CI: .198, .406) which corresponded to moderate stability. BMI *z*-score was tracked with the highest correlation of *r* = .889 (95% CI: .865, .914). This was similar for girls and boys (Figure 3).

Correlations of the overall MP score and MP components of CON were consistently lower than those of NO-PAR (Figure 4). However, no statistically significant differences were found. Correlation of BMI *z*-score in CON (*r* = .847; 95% CI: .804, .890) was significantly lower than its counterpart of NO-PAR (*r* = .923; 95% CI: .896, .949) (Fisher's *z* = −3.039, *p* = .002).

### 3.4. Development of MP and BC

Results of mmANOVA (Table 5) indicated that no changes of MP and BMI *z*-score occurred over grades except for stand and reach (girls: *F*(3, 77.8) = 17.32, *p* < .001, partial *η*^2^ = .400; boys: *F*(3, 83) = 21.63, *p* < .001, partial *η*^2^ = .471) and side jump (girls: *F*(3, 77.9) = 3.15, *p* = .030, partial *η*^2^ = .108; boys: *F*(3, 79) = 3.26, *p* = .026, partial *η*^2^ = .110). For the test item stand and reach, the Bonferroni-Holm-adjusted post hoc tests showed significant differences between each grade for both sexes. For girls, significantly higher values were found in 2nd grade compared to 1st grade (mean difference = 3.35, *p* < .001, *p*_adjusted_ = .013) to 3rd grade (mean difference = 5.86, *p* < .001, *p*_adjusted_ = .009) and to 4th grade (mean difference = 1.56, *p* = .001, *p*_adjusted_ = .025). For boys, the highest values were found at 3rd grade compared to 1st grade (mean difference = 5.89, *p* < .001, *p*_adjusted_ = .010) to 2nd grade (mean difference = 3.90, *p* = .001, *p*_adjusted_ = .017) and to 4th grade (mean difference = 7.58, *p* < .001, *p*_adjusted_ = .009). However, for side jump, the Bonferroni-Holm-adjusted post hoc tests revealed no statistically significant differences between grades.

Children in CON achieved better results than those in NO-PAR in overall MP (refer to Figure 1) (girls: *F*(1, 90.5) = 5.26, *p* = .024, partial *η*^2^ = .055; boys: *F*(1, 84.9) = 21.83, *p* < .001, partial *η*^2^ = .205), standing long jump (girls: *F*(1, 94) = 6.29, *p* = .014, partial *η*^2^ = .063; boys: *F*(1, 92.9) = 16.14, *p* < .001, partial *η*^2^ = .015), and push-ups (girls: *F*(1, 85.1) = 4.86, *p* = .030, partial *η*^2^ = .054; boys: *F*(1, 91.3) = 4.95, *p* = .029, partial *η*^2^ = .052). In addition, boys in CON achieved better results than their counterparts in NO-PAR in stand and reach (*F*(1, 84.7) = 8.82, *p* = .004, partial *η*^2^ = .094), sit-ups (*F*(1, 87.2) = 8.43, *p* = .005, partial *η*^2^ = .088), 6-minute run (*F*(1, 102.8) = 23.52, *p* < .001, partial *η*^2^ = .186), and 20 m sprint (*F*(1, 92.3) = 15.95, *p* < .001, partial *η*^2^ = .147). No statistically significant effects were found in relation to the test item backward balancing and to BMI *z*-score (refer to Figure 2). No statistically significant interaction effects were found.

Table 6 summarizes the results of robust ANCOVA. Positive amounts of the trimmed mean difference indicate higher values of MP and BC for CON. Negative amounts indicate the opposite. Statistical significance of mean differences is given if there is no zero value within the 95% CI.

After adjusting for baseline, boys in CON showed a higher overall MP score than boys in NO-PAR (mean difference = 3.95; 95% CI =2.04, 5.86; *η*^2^ = .028). In addition, boys in CON scored higher on all MP test items except backward balancing and BMI *z*-score. However, the resulting effect size (*η*^2^) was rather small. For girls, robust ANCOVA revealed no statistically significant differences of MP and BMI *z*-score between CON and NO-PAR.

## 4. Discussion

The present study investigated the relationship between MP development and SC participation in German primary school-aged children. The objectives focused on the prevalence of SC participation and the stability of MP and BC and their longitudinal associations with SC participation.

### 4.1. Prevalence of SC Participation

Chi-square tests showed the highest participation rate in CON (56.6%) and the lowest rate in NO (11.2%). This is consistent with other longitudinal studies. Vandorpe et al. [30] reported participation rates of *N* = 301 Flemish children aged 6-9 years with frequencies of 69.8% in CON and 15.0% in NO. Vella et al. [36] reported organized sports participation within an Australian sample (*N* = 3899, aged 10-12 years) showing participation rates of 55.5% in CON (labeled as “participants”) and 14.8% in NO (labeled as “nonparticipants”). On the other hand, our results did not correspond with SC participation rates in a German reference sample [37], in which 6- to 10-year-old children aged 12 to 16 years had lower rates of 48.5% in CON (labeled as “maintenance”) and higher rates of 18.7% in NO (labeled as “nonparticipation”). In contrast to our design, the classification of children into SC participation groups in the abovementioned studies was based on a smaller number of measurement points over a longer period of time, which significantly limits data comparison. Moreover, sociocultural and geocultural factors across countries affect the characteristics of children's PA [38], which may lead to different sports participation rates regardless of the study design. However, consistent participation in SC provides an environment for regular PA that encourages frequent exercise and thus favors the development of children's motor potential. Regular PA can lead to increased PF, reduced body fat, improved bone health, and positive effects on mental components [39]. Regular exercise is a preferred approach to increase self-efficacy, self-esteem, and body awareness [40].

We also found that more girls (38.5%) than boys (24.6%) commenced a SC membership or dropped out during primary school period and thus partially participated in SC. It is not entirely clear, but in terms of participation in SC, girls are more likely to focus on fun or social experiences or have a more appearance-based focus, e.g., body shape [41], than boys. This gender perspective should be taken into account when young girls want to practice their favorite sport in a SC. Furthermore, it seems to be equally important for children who are nonparticipants in SC, which was 13% of girls and 9% of boys in our study (Table 2). However, participation in organized sports in childhood is positively related to PA [42, 43] and can influence the development of healthy habits in adulthood [44].

### 4.2. Tracking of MP and BC

In our study, the stability of MP measured by an overall score was moderately high (*r* = .755). It was slightly higher than the results of Werneck et al. [21] who carried out a 3-year follow-up in 372 children aged 7 to 10 years at baseline. Using a summarized *z*-score of PF components, they reported moderate stability of PF based on Lin's concordance correlation coefficient (girls: LC = .534, boys: LC = .591) and low stability for PF based on kappa coefficient (girls: *κ* = .355, boys: *κ* = .441). Additionally, Lima et al. [13] have tracked gross motor coordination within a subgroup of 515 children matured from 6 to 9 years. The results have shown a moderately high stable motor quotient based on a correlation coefficient of *r* = .62 for both sexes. Assuming negligible sample effects on the range of tracking coefficients in these studies, the choice of tracking method appears to have a large influence on stability. However, given the moderately high stability patterns identified in our study, the course for children's MP appears to have been set at the age of school entry. Therefore, interventions to promote children's motor potential should already be initiated at the beginning of primary school.

Furthermore, we found the highest correlation for BMI *z*-score. We assume that the developmental leap in body height and body mass during primary school years [45] outweighs the effects of other factors that influence BC, such as diet or PA, leading to their high stability. With the lowest correlation for push-ups (*r* = .302), we confirm the results of Roth et al. [14], who reported Pearson's correlation coefficients between grades (.235 ≤ *r* ≤ .414) for two independent longitudinal primary school cohorts (*N* = 252‐315). Beyond instructor-induced measurement error [14], the push-up task is a MP test of muscular endurance but is associated with strength. At the beginning of the empirical study phase, we observed that the performance of push-ups resembled the strenuous performance of a maximum strength test, especially in inexperienced first graders. This latent bias could have affected the number of repetitions and decreased the tracking coefficient.

Our result of high stability of agility (side jump, *r* = .652) slightly differs from previous studies in 6-9-year-old children (*r* = .58 [13]), in 6-10-year-old children (.268 ≤ *r* ≤ .583 [14]), or in 6-12-year-old children (.29 ≤ *r* ≤ .60 [46]). In previous analyses of the same cohort [47], we found the highest absolute rates in the side jump task. Similar to the high stability of BMI *z*-score, this may have resulted in an overcompensation of potential effects of other factors influencing agility performance, so that children's rank positions were largely maintained over time.

The only difference in stability between SC participation groups was determined for BMI *z*-score with a significantly lower *r* in CON. These greater ups and downs in ranking over four years in CON may result from practicing different sports in SC, which we did not observe in this study. Depending on participation in high- or low-impact sports, we know that training is associated with both a decrease in fat mass and an increase in lean mass [48], which directly influence the change in body mass and thus the level of BMI. In addition, Witkowski et al. [49] found significant positive interrelationships between body mass and the direct BC components lean mass, skeletal muscle mass and water content. Despite the reported high stability of BMI *z*-score, this is important information from a public health perspective, as SC participation could help to shift children's weight status into a healthy zone [50].

The lower tracking coefficients in CON for all MP components except cardiorespiratory endurance (6-minute run) suggest that ongoing SC participation is associated with a more unstable course of MP development compared with NO-PAR. An increase of rank position is desirable both for health enhancement and for the development of sport-specific skills. A change to a lower position could provide important information for adjustment training or for initiating additional PA programs. Due to the lack of statistical significance in our study, the lower stability in CON actually remains an assumption that needs more evidence throughout further investigation. In contrast to previous research, which showed higher tracking coefficients for boys [14, 19, 46], there were no statistically significant differences in our study. However, no outcome of confirmatory analysis on the differences was reported in these cited studies. Therefore, we assume similar stability patterns in motor performance in healthy boys and girls.

### 4.3. Development of MP and BC

#### 4.3.1. Within-Subject Development of MP and BC

Robust mmANOVA revealed no time effects of MP and BC except for the MP components flexibility (stand and reach) and agility (side jump). For both sexes, the mean *z*-values of flexibility differed when comparing each grade level. The highest score for girls was obtained in 2nd grade and boys in 3rd grade. The course of flexibility in our study did not represent a linear trend but rather showed year-to-year fluctuations. In contrast, Ruedl et al. [51] showed that sex- and age-standardized flexibility performance appeared to remain at the same level during primary school. However, causes for variation may include a reduction of stretching exercises during PE lessons at participating schools and a focus on training of other MP components like speed or endurance. It should be noted that even in childhood, the absence of flexibility training is associated with an increase in joint stiffness [52]. For agility, the results were inconsistent while post hoc analyses did not confirm the omnibus effect of mmANOVA.

#### 4.3.2. Associations of SC Participation and MP

Children who consistently participated in SC were better in overall MP and the test tasks standing long jump and push-ups. Additionally, only boys showed better MP in 20 m sprint, stand and reach, sit-ups, and 6-minute run. Children's MP benefits from consistent SC participation have been confirmed by other longitudinal studies related to motor coordination in 6- to 9-year-old children [30] and coordination- and physical condition-related MP in 9- to 20-year-old participants [53]. It is widely accepted that better MP is related to higher volumes of moderate and vigorous PA within regular training in organized sports participation [25, 54]. In addition to the intensity of PA during SC participation, the level of sports performance could be another crucial variable contributing to the variance of MP components. When examining the motor potential of experienced judo athletes [49], athletes in the champion class showed significantly better scores in strength endurance and running speed, while athletes from lower sports performance classes achieved significantly higher scores in the explosive strength or flexibility.

Moreover, our investigation revealed no benefit of CON for the MP components balance (backward balancing) and agility (side jump). We assume that these MP components benefit more from engagement in other PA domains, such as extracurricular sports activity or unorganized playing outside. Our findings therefore suggest that it is important to include more coordination-related exercises into the training routine during organized sports participation in order to enhance overall motor potential.

Based on the effect size, cardiorespiratory endurance showed the greatest benefit from CON (boys: *η*^2^ = .182, large; girls: *η*^2^ = .029, small). This was also evident in boys after adjusting for baseline (*η*^2^ = .015, small). This is consistent with Riso et al. [55], who conducted a PREFIT measure in preschool children (*N* = 256, 6.6 ± 0.5 years) and identified significant differences exclusively in cardiorespiratory fitness associated with SC participation. We believe that this is important information because increased levels of cardiorespiratory endurance promote moderate to vigorous PA and may help to fulfill international PA recommendations [56–58].

Robust ANCOVA confirmed higher MP in boys exposed to CON with small effect sizes but revealed no statistically significant differences for girls. Our findings are supported by a longitudinal study of primary school children (*N* = 1067, 6 to 12 years) by Vallence et al. [59] who determined reduced effect sizes for the development of coordination-related, condition-related, and total MP in different organized sports after adjusting for baseline 30 months earlier.

However, the power of the ANCOVA showed significantly higher side jump performance among the boys in CON, which was not evident without baseline adjustment. This implies a small but relevant SC participation benefit for agility as a coordination-related MP component [4] which is an essential prerequisite for the development of peak performance, especially in ball sports [59]. Therefore, from a talent development perspective in elite sports, it is crucial to support children in maintaining their SC participation.

#### 4.3.3. Associations of SC Participation and BC

BMI *z*-score had the same level across the entire sample. Unadjusted analysis showed no effect, and adjustment for baseline showed a small effect size (girls: *η*^2^ = .042, boys: *η*^2^ = .027). Note that in our study, ANCOVA revealed that girls in CON had a higher level and boys a lower level of BMI *z*-score but no statistically significant differences among SC participation (Table 6). In contrast, in annual trend analyses of *N* = 3293 students aged 6 to 14 years, Drenowatz et al. [24] showed that children in SC were significantly associated with lower BMI percentile in each grade, with a more pronounced difference after age 10 years. However, Neil-Sztramko et al. [60] revealed in a systematic review that PA interventions in the school setting led to a very small decrease in BMI *z*-scores. In line with longitudinal analyses of motor coordination by Vandorpe et al. [30], we found no interaction effects for MP or BMI *z*-scores between the independent factor SP participation and the dependent factor grade.

### 4.4. Limitations and Strengths

Some limitations should be noted when interpreting the results of our study. SC participation was measured annually, so changes in membership between occasions were unknown and therefore may have influenced a consistent matching of subjects to independent groups. Merging the NO and PAR groups was a compromise to obtain sufficiently large subsamples and at least two contrasting groups. Following Telford et al. [25], no subject should be excluded, and the three patterns of participation should be considered [30]. However, our study focused only on self-reported membership in a SC. Neither the type of sport nor the duration or frequency of training sessions were included in the data collection. Consistent with the assessment of MP using DMT 6-18 by Roth et al. [14], test instructors were trained but changed throughout the study. Furthermore, due to similar motor tasks in PE, we cannot exclude a systematic practice effect on the test procedure. These aspects may have led to an unknown bias in the level and development of MP.

On the other hand, the use of the DMT 6-18 as a valid and reliable test battery for the population of children [4] represents a strength of the study. Processing raw data into standardized MP values permits the consideration of age- and sex-specific influences and reduces growth-related influences due to variations in maturation and puberty. Since it was possible that children with higher MP scores were more likely to participate in SC, we additionally included the baseline measure as a covariate. Thus, systematic overestimation of MP, BC, and SC participation group effects can be reduced. The KOMPASS-2-study, together with other cross-regional studies, is part of the representative data set of the Germany's 2022 Report Card on Physical Activity survey [61]. In this regard, our results are generalizable to children in Germany.

## 5. Conclusions

Despite sufficient evidence of the positive effects of participation in organized sports on health, our study found over 40% of children only partially or never joined SCs. Girls, in particular, were characterized by discontinuous SC participation, which was reflected in more frequent dropout or later commence of SC membership during primary school years. Therefore, further SC participation strategies should allow children, and especially girls, to change not only between different SCs but also within a SC to ensure consistent participation and sustain training. Interventions to increase engagement in SC should aim for a stepwise change in behavior, e.g., from no to partial SC participation, from partial to consistent SC participation, and from short-term to long-term maintenance of SC participation. Conducting tracking analyses during such a stepwise change would result in a desirably unstable course of PA and associated MP. Therefore, when interpreting tracking results in further studies, not only single stability coefficients but also the direction of change of an individual's position should be taken into account.

Our findings revealed that children who consistently participated in SC over a four-year period exhibited enhanced MP, with the greatest benefit observed in cardiorespiratory endurance. Cardiorespiratory endurance is an important health resource and a basic prerequisite for achieving peak performance. Beyond children's MP, further research should therefore focus on health- and performance-related outcomes and examine participation patterns in more detail. Finally, to better understand the associations between BC and SC participation, there is a need to replicate studies using direct BC parameters such as fat mass or skinfold thickness. In this regard, other MP assessment approaches such as the ALPHA Fitness Test Battery [6] may seem to be an appropriate alternative for DMT 6-18.

## Figures and Tables

**Table 1:** Descriptive statistics of the children across the measurement time points.

Sex	Grade 1		Grade 2		Grade 3		Grade 4	
	*N*	Age (yrs) *m* ± SD	*N*	Age (yrs) *m* ± SD	*N*	Age (yrs) *m* ± SD	*N*	Age (yrs) *m* ± SD
Girls	579	6.98 ± 0.39	575	7.96 ± 0.39	516	9.00 ± 0.43	504	9.95 ± 0.41
Boys	561	7.02 ± 0.39	560	7.98 ± 0.39	503	9.01 ± 0.39	504	9.99 ± 0.41
Total	1140	7.00 ± 0.39	1135	7.97 ± 0.39	1019	9.01 ± 0.41	1008	9.97 ± 0.41

Note: *m* = mean; SD = standard deviation; yrs = years.

**Table 2:** Number and percentage of children in relation to SC participation.

	CON	NO	PAR	Total
	*N* (%)	*N* (%)	*N* (%)	*N*
Girls	78 (48.4)	21 (13.0)	62 (38.5)	161
Boys	89 (66.4)	12 (9.0)	33 (24.6)	134
Total	167 (56.6)	33 (11.2)	95 (32.2)	295

Note: CON = consistent SC participation; NO = no SC participation; PAR = partial SC participation.

**Table 3:** Descriptive statistics of MP components in SC participation across sex and grade (sex- and age-standardized values: mean ± *s*).

Test items	Sex	Sports club	Grade 1 (6.9 ± 0.4 yrs)		Grade 2 (7.9 ± 0.4 yrs)		Grade 3 (8.9 ± 0.4 yrs)		Grade 4 (9.9 ± 0.4 yrs)	
Stand and reach	Girls	CON	106.03	10.23	108.14	9.81	102.27	5.06	106.83	9.63
		NO-PAR	102.28	10.08	106.87	9.96	101.01	6.33	105.05	9.90
	Boys	CON	96.35	9.74	98.96	8.84	102.17	5.49	95.16	9.46
		NO-PAR	91.89	10.69	93.27	10.24	97.84	6.63	89.69	10.30

Side jump	Girls	CON	101.86	9.03	101.72	7.92	101.99	8.23	101.91	8.04
		NO-PAR	99.57	9.98	100.30	9.79	101.07	10.15	99.60	9.68
	Boys	CON	103.28	9.32	103.18	8.78	102.60	9.07	103.88	8.99
		NO-PAR	101.04	9.14	100.42	10.01	97.38	9.95	100.16	10.06

Sit-ups	Girls	CON	101.65	9.36	100.42	9.03	101.64	7.35	101.74	9.01
		NO-PAR	98.12	10.91	98.06	9.83	98.81	10.61	99.86	10.55
	Boys	CON	102.26	9.50	105.39	9.00	102.73	8.94	105.37	9.79
		NO-PAR	100.09	9.81	99.49	8.35	99.02	9.71	100.29	10.17

6-minute run	Girls	CON	101.29	8.14	101.50	8.72	100.81	8.26	100.65	8.47
		NO-PAR	98.90	8.76	99.16	10.88	99.77	11.47	99.36	10.01
	Boys	CON	104.36	7.43	104.44	8.37	103.01	9.66	104.80	8.68
		NO-PAR	99.20	6.71	99.22	7.61	97.82	8.76	97.11	10.17

20 m sprint	Girls	CON	102.37	8.85	102.67	8.18	101.21	8.73	102.19	9.32
		NO-PAR	100.72	8.62	100.23	8.95	101.57	9.92	100.35	9.86
	Boys	CON	103.80	7.83	104.57	8.47	102.89	8.43	103.96	7.86
		NO-PAR	98.04	9.64	98.98	10.09	97.07	11.04	97.73	11.06

Standing long jump	Girls	CON	103.08	9.56	102.82	8.69	101.60	9.60	101.92	8.84
		NO-PAR	99.42	9.24	99.23	9.84	99.71	8.68	98.42	9.26
	Boys	CON	104.48	10.13	102.81	10.26	103.11	9.10	102.72	8.54
		NO-PAR	100.09	10.57	99.24	10.82	96.87	10.57	98.93	11.51

Backward balancing	Girls	CON	101.22	8.41	102.67	8.21	101.18	9.78	101.13	9.93
		NO-PAR	99.84	9.81	100.27	9.07	100.33	10.27	100.29	9.39
	Boys	CON	103.22	9.95	101.63	9.16	101.52	9.48	102.61	8.50
		NO-PAR	99.78	10.03	99.98	10.32	99.24	10.72	98.27	10.17

Push-ups	Girls	CON	102.87	8.87	103.09	9.12	101.37	8.46	101.45	8.40
		NO-PAR	99.36	11.19	98.89	8.69	98.93	10.85	100.65	11.34
	Boys	CON	102.45	8.27	103.87	9.64	102.26	9.33	104.16	9.60
		NO-PAR	100.67	10.07	100.07	7.18	100.38	9.48	100.36	8.45

Note: CON = consistent SC participation; NO-PAR = no and partial SC participation.

**Table 4:** Tracking of MP and BC using mean Pearson's correlation coefficient (*r*).

Test items	*r*	95% CI		Stability [10]
		Lower	Upper	
BMI *z*-score	0.889	0.865	0.914	Moderately high
Side jump	0.652	0.586	0.718	Moderately high
Sit-ups	0.611	0.539	0.683	Moderately high
Stand and reach	0.584	0.508	0.660	Moderate
Standing long jump	0.557	0.478	0.636	Moderate
20 m sprint	0.547	0.466	0.627	Moderate
6-minute run	0.469	0.379	0.558	Moderate
Backward balancing	0.434	0.341	0.528	Moderate
Push-ups	0.302	0.198	0.406	Moderate
Overall MP score	0.755	0.706	0.804	Moderately high

**Table 5:** Robust mmANOVA results of sex- and age-standardized values of MP and BC for the longitudinal sample (*N* = 295).

Test item	Effect	Girls (*N* = 161)					Boys (*N* = 135)				
		*F*	df_1_	df_2_	*p*	*η* ^2^	*F*	df_1_	df_2_	*p*	*η* ^2^
BMI *z*-score	Grade	0.92	3	76.0	.434	.035	0.06	3	85.3	.983	.002
	Sports club	0.24	1	93.9	.623	.003	0.50	1	81.1	.480	.006
	Sports club × grade	1.27	3	76.0	.292	.064	0.38	3	85.3	.767	.013

Stand and reach	Grade	17.32	3	77.8	<.001	.400	21.63	3	83.0	<.001	.471
	Sports club	3.14	1	93.4	.080	.033	8.82	1	84.7	.004	.094
	Sports club × grade	1.41	3	77.8	.246	.052	0.05	3	83.0	.985	.002

Side jump	Grade	3.15	3	77.9	.030	.108	3.26	3	79.0	.026	.110
	Sports club	1.86	1	89.1	.176	.020	3.24	1	87.0	.075	.036
	Sports club × grade	0.67	3	77.9	.573	.025	1.21	3	79.0	.310	.044

Sit-ups	Grade	1.31	3	76.8	.277	.049	1.85	3	83.1	.145	.063
	Sports club	3.77	1	90.8	.055	.040	8.43	1	87.2	.005	.088
	Sports club × grade	0.73	3	76.8	.538	.028	1.79	3	83.1	.155	.061

6-minute run	Grade	0.23	3	77.3	.878	.009	2.59	3	80.2	.058	.088
	Sports club	2.77	1	92.5	.099	.029	23.52	1	102.8	<.001	.186
	Sports club × grade	0.71	3	77.3	.551	.027	2.20	3	80.2	.095	.076

20 m sprint	Grade	0.46	3	77.8	.709	.017	1.53	3	79.9	.214	.054
	Sports club	1.52	1	92.9	.221	.016	15.95	1	92.3	<.001	.147
	Sports club × grade	1.64	3	77.8	.188	.060	0.05	3	79.9	.983	.002

Standing long jump	Grade	0.95	3	77.7	.421	.035	1.22	3	79.0	.308	.043
	Sports club	6.29	1	94.0	.014	.063	16.14	1	92.9	<.001	.015
	Sports club × grade	1.81	3	77.7	.151	.065	0.90	3	79.0	.445	.033

Backward balancing	Grade	1.45	3	77.5	.235	.053	0.11	3	80.3	.957	.004
	Sports club	1.36	1	93.5	.247	.014	3.14	1	85.1	.080	.036
	Sports club × grade	0.28	3	77.5	.839	.011	0.81	3	80.3	.492	.029

Push-ups	Grade	0.46	3	77.6	.708	.018	0.28	3	85.3	.842	.010
	Sports club	4.86	1	85.1	.030	.054	4.95	1	91.3	.029	.052
	Sports club × grade	1.26	3	77.6	.295	.046	1.19	3	85.3	.317	.040

Overall MP score	Grade	0.29	3	77.6	.835	.011	2.44	3	79.2	.070	.085
	Sports club	5.26	1	90.5	.024	.055	21.83	1	84.9	<.001	.205
	Sports club × grade	2.02	3	77.6	.117	.072	0.23	3	79.2	.878	.009

**Table 6:** Robust ANCOVA results of sex- and age-standardized values of MP and BC among SC participation for the longitudinal sample (*N* = 295) considering baseline data as covariate.

Test item	Girls (*N* = 161)					Boys (*N* = 135)				
	*m*_Difference_	95% CI		*t*_y_	*η* ^2^	*m*_Difference_	95% CI		*t*_y_	*η* ^2^
		Lower	Upper				Lower	Upper		
BMI *z*-score	6.14	-1.16	13.43	2.41	.042	-5.35	-14.49	3.80	1.81	.027
Stand and reach	5.06	-0.88	11.01	2.32	.016	4.00	0.95	7.05	3.40	.012
Side jump	-3.16	-7.31	0.99	2.00	.006	6.62	0.04	13.19	2.77	.040
Sit-ups	-6.69	-16.07	2.69	2.05	.019	5.88	2.98	8.78	5.30	.028
6-minute run	-11.94	-28.51	4.63	2.19	.032	5.09	1.41	8.78	3.67	.015
20 m sprint	-3.07	-13.70	7.55	0.90	.003	2.73	0.21	5.25	2.83	.008
Standing long jump	-2.23	-6.66	2.21	1.35	.005	6,63	3.72	9.55	5.90	.025
Backward balancing	-1.83	-5.07	1.42	1.45	.002	2.81	-0.97	6.60	1.94	.004
Push-ups	-5.18	-14.64	4.29	1.75	.008	3.22	0.50	5.94	3.07	.007
Overall MP score	-1.43	-3.51	0.65	1.82	.009	3.95	2.04	5.86	5.41	.028

*m*_Difference_ = trimmed mean difference; *t*_y_ = Yuen's *t*-value.

## Data Availability

The empirical data used to support the findings of this study are included within the article. All underlying data of this study are available from the corresponding author upon request.
